# Synergistic application of pulmonary ^18^F-FDG PET/HRCT and computer-based CT analysis with conventional severity measures to refine current risk stratification in idiopathic pulmonary fibrosis (IPF)

**DOI:** 10.1007/s00259-019-04386-5

**Published:** 2019-07-08

**Authors:** Francesco Fraioli, Maria Lyasheva, Joanna C. Porter, Jamshed Bomanji, Robert I. Shortman, Raymond Endozo, Simon Wan, Linda Bertoletti, Maria Machado, Balaji Ganeshan, Thida Win, Ashley M. Groves

**Affiliations:** 1 Institute of Nuclear Medicine, UCL(H) and University College London Hospital, 235 Euston Rd, London, NW1 2BU UK; 2 Department of Oncology, School of Clinical Medicine, University of Cambridge, Cambridge, UK; 3 CITR, UCL and Interstitial Lung Disease Centre, UCLH, London, UK; 4 Imaging Department, University of Rome “Sapienza”, Rome, Italy; 5 Respiratory Medicine, Lister Hospital, Stevenage, UK

**Keywords:** Idiopathic pulmonary fibrosis, Quantitative computer analysis, Positron emission tomography, Fluorine-18 FDG and pulmonary vessels

## Abstract

**Introduction:**

To investigate the combined performance of quantitative CT (qCT) following a computer algorithm analysis (IMBIO) and ^18^F-FDG PET/CT to assess survival in patients with idiopathic pulmonary fibrosis (IPF).

**Methods:**

A total of 113 IPF patients (age 70 ± 9 years) prospectively and consecutively underwent ^18^F-FDG PET/CT and high-resolution CT (HRCT) at our institution. During a mean follow-up of 29.6 ± 26 months, 44 (48%) patients died. As part of the qCT analysis, pattern evaluation of HRCT (using IMBIO software) included the total extent (percentage) of the following features: normal-appearing lung, hyperlucent lung, parenchymal damage (comprising ground-glass opacification, reticular pattern and honeycombing), and the pulmonary vessels. The maximum (SUV_max_) and minimum (SUV_min_) standardized uptake value (SUV) for ^18^F-FDG uptake in the lungs, and the target-to-background (SUV_max_/SUV_min_) ratio (TBR) were quantified using routine region-of-interest (ROI) analysis. Pulmonary functional tests (PFTs) were acquired within 14 days of the PET/CT/HRCT scan. Kaplan–Meier (KM) survival analysis was used to identify associations with mortality.

**Results:**

Data from 91 patients were available for comparative analysis. The average ± SD GAP [gender, age, physiology] score was 4.2 ± 1.7 (range 0–8). The average ± SD SUV_max_, SUV_min_, and TBR were 3.4 ± 1.4, 0.7 ± 0.2, and 5.6 ± 2.8, respectively. In all patients, qCT analysis demonstrated a predominantly reticular lung pattern (14.9 ± 12.4%). KM analysis showed that TBR (*p* = 0.018) and parenchymal damage assessed by qCT (*p* = 0.0002) were the best predictors of survival. Adding TBR and qCT to the GAP score significantly increased the ability to differentiate between high and low risk (*p* < 0.0001).

**Conclusion:**

^18^F-FDG PET and qCT are independent and synergistic in predicting mortality in patients with IPF.

## Introduction

High-resolution CT (HRCT) is the current imaging reference standard in the investigation of patients with idiopathic pulmonary fibrosis (IPF), revealing structural details of the entire lung parenchyma which reflect the characteristic histological changes. However, HRCT is conventionally a purely structural, qualitative imaging technique and requires dedicated radiological training to assess the severity of disease [1, 2]. Several computer-based quantitative CT (qCT) methods have been developed to precisely quantify the extent of disease [3]. However, despite showing a significant correlation with both visual score and pulmonary function tests (PFTs), qCT has yet to provide insight into the mechanisms and activity of fibrosis and disease progression. The ability to radiologically quantify the response of individual patients to treatment would be an important advance given the current lack of end points for drug trials in IPF [4].

Positron emission tomography (PET) offers the means for non-invasive investigation of cellular metabolism in vivo. PET studies in animals have yielded potentially valuable insight into the biology of IPF, with heightened ^18^F-fluorodeoxyglucose (^18^F-FDG) PET signal intensity related to interstitial lung changes [5, 6]. ^18^F-FDG pulmonary uptake on PET has been shown to relate to disease severity as assessed by quality-of-life measurement, lung volume, and gas transfer, and more recently it was shown that baseline objective measures of ^18^F-FDG uptake on PET predict patient survival, independent of PFTs [7, 8].

Given the need for biomarkers in patients with IPF for risk stratification and drug development, we investigated the potential synergistic application of pulmonary^18^F-FDG PET signal with qCT to predict survival in IPF. We compared this synergistic approach with the clinical gender-age-physiology (GAP) scoring system for prognostic accuracy [9].

## Materials and methods

From January 2008 to December 2017, a total of 113 (93 male, 20 female, mean age 70 ± 8.9 years) prospective and consecutive patients with IPF underwent ^18^F-FDG PET/CT/HRCT at our institution. The entire cohort was previously reported in a study assessing only FDG PET [8]. In the present study, a subgroup of that cohort is used for additional analysis (qCT) to compare and explore the synergistic value of qCT as compared to the previously reported FDG PET findings. All patients underwent full clinical assessment and baseline pulmonary function tests (PFTs).

All patients were referred from primary and secondary care to the UCLH NHS-England Specialist Centre for the diagnosis and management of interstitial lung diseases (ILDs). The mean average time from first visit to our tertiary care center was 15 ± 10 months (4–23 months), and patients displayed a wide variation in levels of dyspnea when first assessed.

Patients with symptoms of acute infection and lung malignancy were excluded. Diagnosis of IPF was made on clinical and radiological grounds following multidisciplinary team (MDT) review. The MDT comprises specialists including at least two ILD-trained radiologists, three specialist ILD respiratory physicians, a specialist nurse and a lung pathologist. A dedicated clinical assessment and investigations were used to rule out other possible causes of usual interstitial pneumonitis (UIP) that can give the same radiological picture as IPF. This includes other fibrosing ILDs such as hypersensitivity pneumonitis, sarcoidosis, and rheumatoid arthritis.

The study was approved by the ethics board (London-Harrow Research Ethics Committee [REC reference 06/Q0505/22]), and all patients provided written informed consent.

The follow-up period was defined from the date of scan to death (all causes) or 9 years, whichever happened first. Repeat scans were performed when clinically indicated, and not routinely unless this affected patient management, according to National Institute for Health and Care Excellence (NICE) IPF clinical guidelines.Patient survival was confirmed by the use of patient charts, electronic database, primary health care physician records, or telephone interview.

The GAP index was computed based on four variables: gender (G), age (A), and two lung physiology (P) parameters, forced vital capacity (FVC) and transfer factor of the lung for carbon monoxide (TLCO) [10]. This comprised a model using continuous predictors (GAP calculator) and a simple point-scoring system (GAP index), which varies from 0, potentially indicating a good outcome, to 8, potentially indicating a worse outcome.

Based on the GAP index, the three stages identified are as follows: GAP stage I included GAP index 0, 1, 2, 3; GAP stage II included GAP index 4, 5; and GAP stage III included GAP index 6, 7, 8.

### PET/CT acquisition

The PET/CT scans were obtained using a 64-slice multidetector CT scanner (VCT PET/64, GE Healthcare, Chicago, IL, USA).

Three imaging sequences of the thorax were performed while the patient remained supine on the table throughout. A CT scan was performed for attenuation correction. With the patient maintaining the supine position, a chest ^18^F-FDG PET emission scan (8 min/bed position) was performed 1 h after injection of 200 MBq of ^18^F-FDG.

After completion of the PET/CT, with the patient maintaining the same supine position, an HRCT (volumetric 1-mm full inspiration scan, peak voltage of 120 kVp, tube current modulation range 30–140 mA, B70 kernel reconstruction) was performed.

### Image display and processing

PET/CT images were analyzed by a dedicated thoracic radiologist and senior PET technologist with more than 10 years of experience in quantifying pulmonary ^18^F-FDG PET uptake in ILD and examining HRCT.

All images were loaded onto a dedicated workstation. Using a volumetric region of interest, the area of most intense pulmonary ^18^F-FDG uptake was identified and the highest value (SUV_max_) measured. In addition, the region of pulmonary parenchyma considered mostly normal on CT by the expert thoracic radiologist, with the lowest SUV, was identified and the SUV_min_ in this region recorded. The target-to-background ratio (TBR = SUV_max_/SUV_min_) was also recorded as a standard measurement [11].

QCT analysis on HRCT was undertaken using IMBIO software (the technical features have been described [12] previously). Briefly, evaluation of HRCT data involved algorithmic identification and volumetric quantification of every voxel volume unit into one of the following radiologic parenchymal features: normal-appearing lung, hyperlucent lung, ground-glass opacification, reticular pattern, honeycombing, and the pulmonary vessels. Volumes were then converted into percentages using, as reference, the total volume of the lungs as measured by the software. A synthetic value was created by adding up ground-glass opacification, reticular pattern, and honeycombing to express the total burden of disease in the lung parenchyma.

### Statistical analysis

Statistical analyses were performed using RStudio version 1.1.463 (RStudio Inc., Boston, MA, USA) for Macintosh based on R version 3.5.1 (R Foundation for Statistical Computing).

Correlations between the area of highest pulmonary ^18^F-FDG uptake (SUV_max_), lowest pulmonary ^18^F-FDG uptake (SUV_min_), TBR, extent of qCT parenchymal patterns, and individual PFTs were explored with Spearman’s correlation coefficient and displayed using a correlation matrix; to account for multiple comparisons, the Bonferroni correction was applied (*p* < 0.0042).

The survival analysis was performed using the package ‘survminer’ (https://cran.r-project.org/web/packages/survminer/) and its dependencies. To explore the relationships of imaging-derived parameters (PET and qCT), PFTs, and GAP with patient survival, a Kaplan–Meier (KM) survival analysis was calculated for each of the parameters. Patients that were alive at the time of the follow-up collection were censored. Initially, the median value was chosen as a threshold (cutoff) to divide the cohort into two groups according to their prognosis (poor and good prognostic groups). Subsequently, optimal cutoffs that best separated the survival plots were determined (lowest *p* value). KM curves displaying patients above and below each threshold were generated to facilitate the visualization of the survival trend of the two populations. Subsequently, the parameters that were found to significantly discriminate between the prognostic groups were used as input for a multivariate stepwise (forward and backward) Cox regression. The variables to be retained at each step were determined using the Akaike information criterion.

In all the analyses that were not corrected for multiple comparison, *p* values <0.05 were considered significant.

### Modified PET and qCT score

The potential synergistic effect of GAP and imaging-derived biomarkers (PET and qCT) in prognostication was assessed creating a novel score based on the GAP index. We propose to add to the GAP index another factor based on the best predictor for PET (either SUV_max_, SUV_min_ or TBR) according to the prognostic ability of the biomarker to create a synthetic score based on the imaging test as previously described [8] (hereafter called GAP_PET). Similarly, we propose to use the best biomarker derived from the qCT analysis as an added factor for a third modified score (hereafter GAP_PET_qCT). According to the best cutoff point previously determined, imaging biomarkers were binarized in adverse (coded as 1) or favorable (coded as 0) biomarkers. This was subsequently added to the existing GAP index value, resulting in GAP_PET ranging from 0 to 9 and GAP_PET_qCT ranging from 0 to 10, as compared to the standard GAP index, which is from 0 to 8. The new scores redefined the stages as follows: stage I for GAP_PET as 0–3 and for GAP_PET_qCT as 0–4; stage II for GAP_PET as 4–6 and for GAP_PET_qCT as 5–7; and stage III for GAP_PET as 7–9 and for GAP_PET_qCT as 8–10.

## Results

The HRCT was not analyzable by the qCT software in 22 patients. Reasons for failure were motion artifacts in 17 patients and incorrect reconstruction kernel in 5 patients. Thus, 91 patients with IPF were included and analyzed for this comparative FDG PET/qCT study.

Of the retained patients, 78 (85.7%) were male, and 23 (25.3%) were treated with pirfenidone. At baseline, the average GAP index was 4.2 ± 1.7 (0–8); 31 patients (34.4%) were classified as GAP stage I, 38 patients (42.2%) were GAP stage II, and the remaining 21 patients (23.3%) were classified as stage III; one patient was excluded from the GAP analysis because the FVC was unobtainable. Values of FVC, forced expiratory volume in 1 s (FEV1), total lung capacity (TLC), carbon monoxide transfer coefficient (KCO), and TLCO are shown in Table 1. The mean follow-up period was 29.6 ± 26 months (0–109.4); during this time, 47 (51.6%) patients died. Cause of death was as follows: exacerbation of IPF (17 [37%]), pulmonary embolism (3 [6%]), pneumonia (14 [31%]), heart failure/cor polmonale (6 [12%]), 3 cancer (2 lung and 1 colon [6%]), and 4 unknown (8%).

**Table 1:** Pulmonary function tests (PFTs) obtained at baseline

PFTs	Value
FVC	74.9 ± 17.6 (37–122)
FEV1	77.1 ± 16.5 (31.8–129)
TLC	69.9 ± 11.1 (48–91)
KCO	77.8 ± 21.7 (27–135)
TLCO	45.3 ± 14.4 (11–79)
GAP index	4.2 ± 1.7 (0–8)

Figures are expressed as mean ± standard deviation (range). FVC = forced vital capacity, FEV1 = forced expiratory volume in 1 s, TLC = total lung capacity, KCO = carbon monoxide transfer coefficient, TLCO = transfer factor of the lung for carbon monoxide. The GAP index was obtained as per *Ley B, Ryerson CJ, Vittinghoff E, Ryu JH, Tomassetti S, Lee JS, Poletti V, Buccioli M, Elicker BM, Jones KD, King TE Jr, Collard HR. A multidimensional index and staging system for idiopathic pulmonary fibrosis. Ann Intern Med. 2012 May 15;156(10):684-91. doi:*10.7326/0003-4819-156-10-201,205,150-00004*. PubMed PMID: 22586007*)

The mean SUV_max_ was 3.4 ± 1.4 (1.5–10.7), the mean SUV_min_ (background lung activity) was 0.7 ± 0.2 (0.3–1.3), and the mean TBR 5.7 ± 2.8 (2.2–21.4). The results of the qCT analysis are shown in Table 2; the predominant pattern was reticular lung, with an average percentage of 14.9 ± 12.4% (0.3–74.5); on average, 71.8 ± 16% (19.2–94) of lung parenchyma was deemed normal.

**Table 2:** PET values and quantitative CT parameters derived from PET/CT and HRCT

Parameter	Value
PET	
SUV_max_	3.4 ± 1.4 (1.5–10.7)
SUV_min_	0.7 ± 0.2 (0.3–1.3)
TBR	5.7 ± 2.8 (2.2–21.4)
qCT	
Normal parenchyma (%)	71.8 ± 16 (19.2–94)
Normal parenchyma (cm^3^)	2986.1 ± 1236.6 (83.9–5600)
Hyperlucent (%)	5 ± 6.9 (0.00005–26)
Ground-glass (%)	6.3 ± 8.6 (0.04–50.6)
Reticular (%)	14.9 ± 12.4 (0.3 ± 74.5)
Honeycomb (%)	1.8 ± 2.2 (90–9.3)
Parenchymal damage (%)	23 ± 16.7 (0.8–80.8)
Vessels (%)	3.9 ± 1.6 (0.9–9.8)
Vessels (cm^3^)	147.5 ± 47.9 (67.1–304.7)

Values are expressed as mean ± standard deviation (range)

### Bivariate analysis

**Fig. 1:**
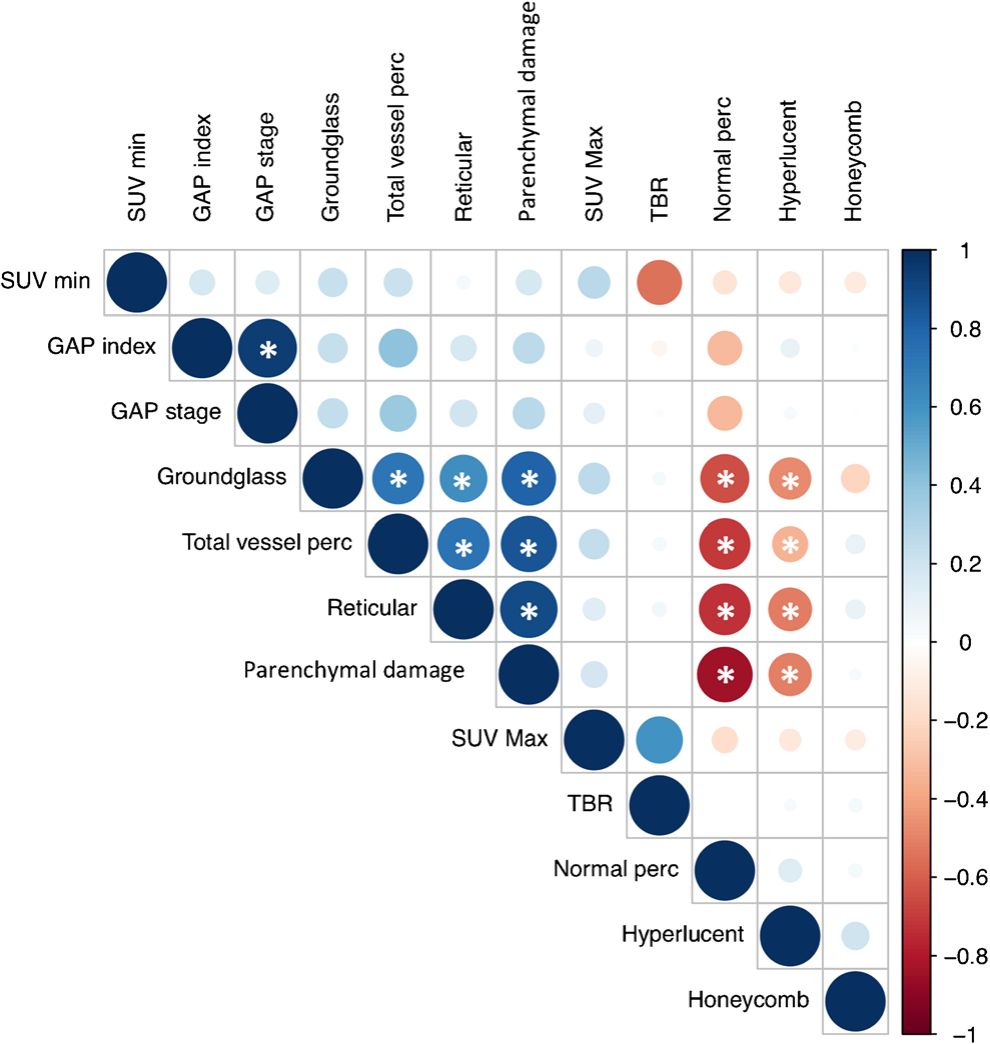
Correlation matrices among the highest pulmonary ^18^F-FDG uptake (SUV_max_), lowest uptake (SUV_min_), TBR, extent of qCT parenchymal patterns, and GAP index and stage. Correlations were explored using Spearman’s correlation coefficient; to account for multiple comparisons, the Bonferroni correction was applied. A value of 1 indicates complete positive correlation; a value of −1 indicates complete negative correlation. *Indicates statistical significance

Correlations between the area with highest pulmonary ^18^F-FDG uptake (SUV_max_), lowest pulmonary uptake (SUV_min_), TBR, extent of qCT parenchymal patterns, and individual PFTs are shown in Fig. 1. The imaging-derived parameters available from the qCT software correlated significantly among themselves (0.0000 < *p* < 0.004), acting as internal validation for the software itself. Of the PFTs, only TLC was correlated with an imaging biomarker (SUV_min_); this correlation was negative (r = −0.76; *p* = 0.0025).

### Univariate and multivariate survival analysis

The KM analysis was performed for all the clinical variables (PFTs and GAP) and the imaging-derived biomarkers (PET and qCT); results are summarized in Table 3.

**Table 3:** Results of Kaplan–Meier analysis conducted using the median values as cutoff to discriminate between the two groups (presence or lack of event during follow-up)

Parameter	Cutoff	*p* value
Pulmonary function tests		
FVC	74	0.01
FEV1	77	0.036
TLC	69	0.21
KCO	78	0.032
TLCO	46	0.0004
GAP index	4	0.018
PET-derived parameters		
SUV_max_	3.1	0.33
SUV_min_	0.6	0.29
TBR	5	0.019
qCT		
Total volume (cm^3^)	4103.86	0.2
Vessel percentage (%)	3.80	<0.0001
Normal parenchyma (%)	74.56	0.011
Hyperlucent (%)	2.05	0.69
Ground-glass (%)	2.80	0.017
Reticular (%)	11.46	<0.0001
Honeycomb (%)	0.71	0.5
Parenchymal damage (%)	18.94	0.0013

FVC = forced vital capacity, FEV1 = forced expiratory volume in 1 s, TLC = total lung capacity, KCO = carbon monoxide transfer coefficient, TLCO = transfer factor of the lung for carbon monoxide

**Fig. 2:**
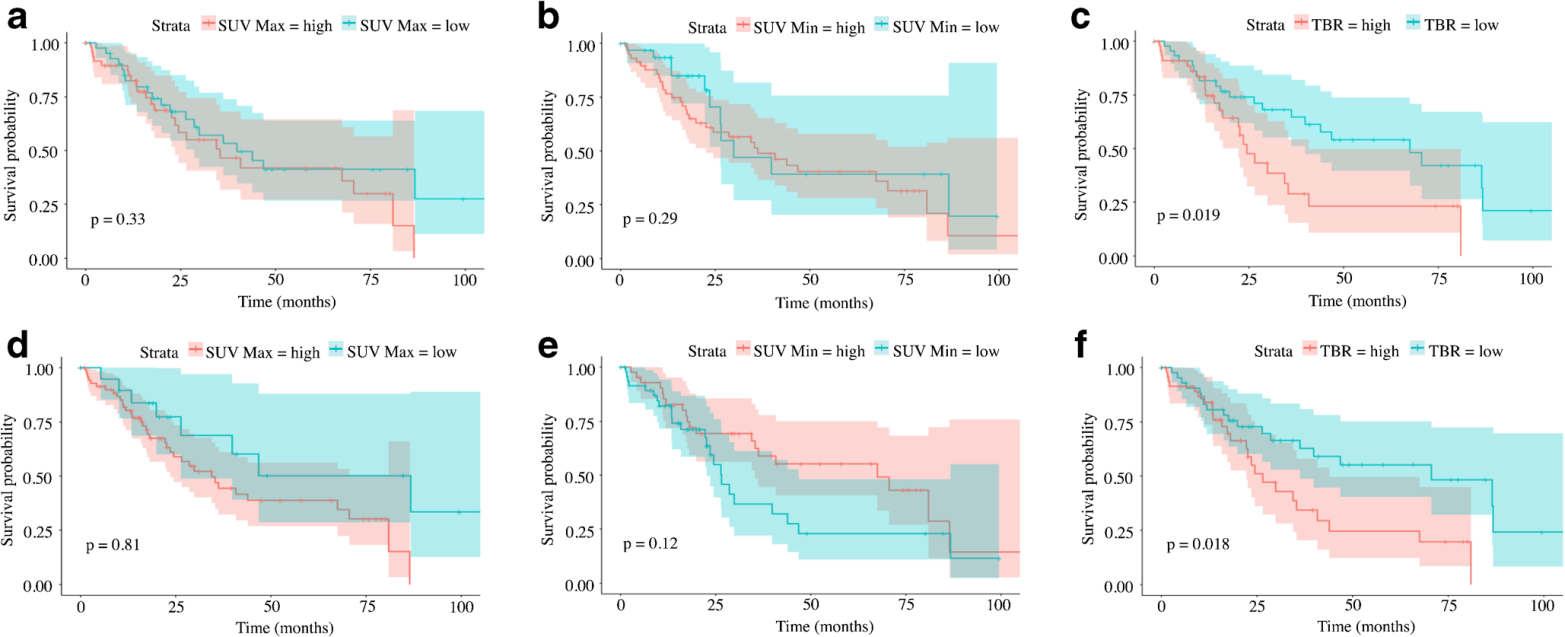
Kaplan–Meier plots of survival analysis. Patients were classified according to their median (*a* is SUV_max_, *b* is SUV_min_, and *c* is TBR) and according to the optimal cutoff (*d* is SUV_max_, *e* is SUV_min_, and *f* is TBR) values as described in the methods section. The log-rank test demonstrated a statistically significant worse prognosis in patients with TBR greater than 5 (median value, *p* = 0.019, curve *c*) and 4.8 (optimal cutoff, *p* = 0.018, curve *f*). Using the optimal cutoff for all the parameters improved the capacity of differentiating between patients with better and worse prognosis; however, SUV_max_ (*a* and *d*) and SUV_min_ (*b* and *e*) were not statistically significant

**Fig. 3:**
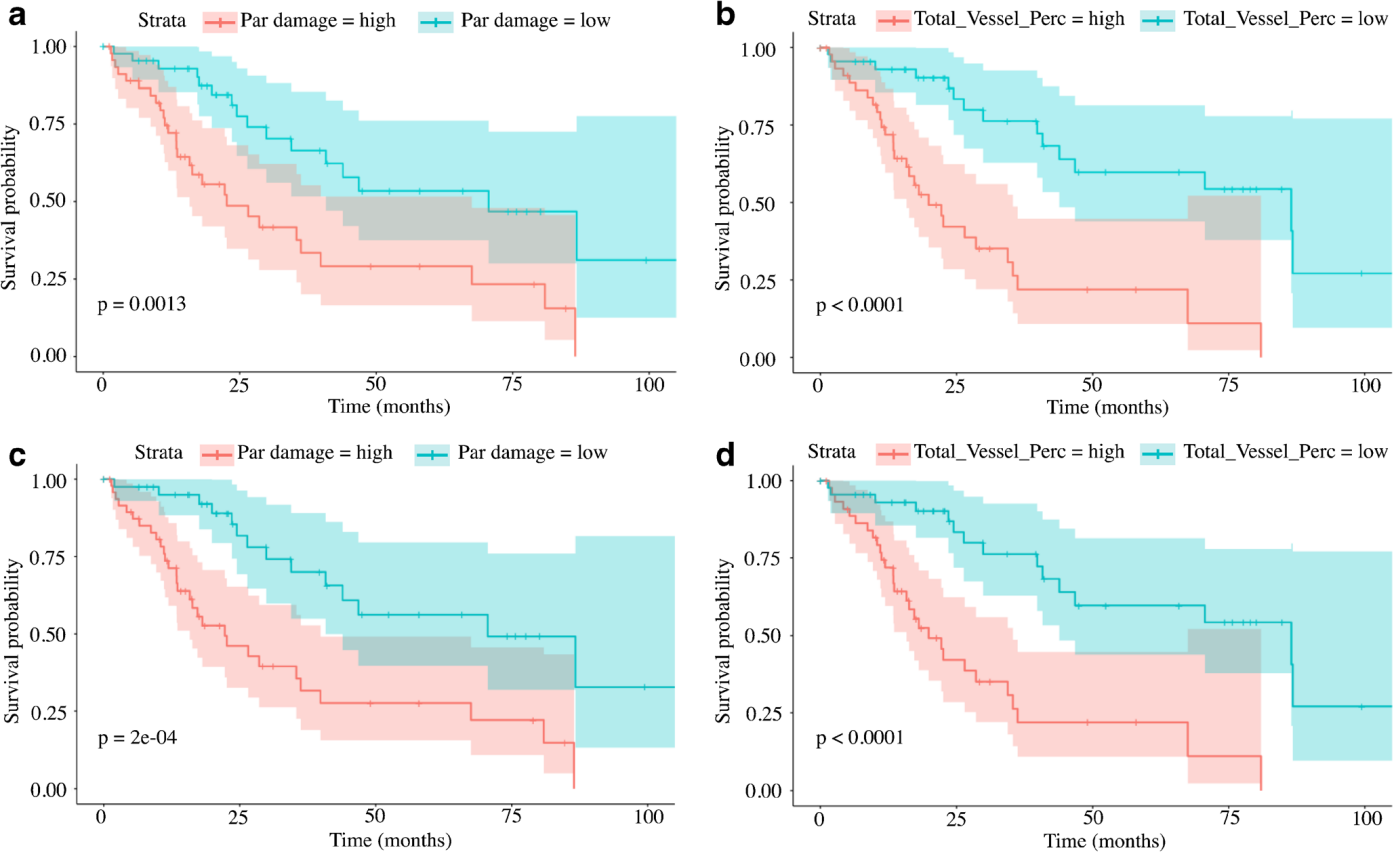
Kaplan–Meier plots of survival analysis. Patients were classified according to their median (*a* is parenchymal damage, *b* is total vessel percentage) and according to the optimal cutoff (*d* is parenchymal damage and *e* is total vessel percentage) values, as described in the methods section. The parenchymal damage was obtained by adding up ground-glass opacification, reticular pattern, and honeycombing to express the total burden of disease in the lung parenchyma; the total vessel percentage reflects the percentage of vessels in the lung parenchyma. The log-rank test demonstrated a statistically significant worse prognosis in patients with parenchymal damage greater than 18.9 (median value, *p* = 0.0013, curve *a*) and 18.5 (optimal cutoff, *p* = 0.0002, curve *c*). Similarly, a vessel percentage greater than 3.8% (median value, *p* < 0.0001, curve *b*) and 3.66% (optimal cutoff, *p* < 0.0001, curve *d*) predicted significantly worse prognosis. Using the optimal cutoff calculated in R improved the capacity to differentiate between patients with better and worse prognosis, using both parenchymal damage and total vessel percentage

The KM analysis using the median as cutoff value demonstrated that all the PFTs with the exception of the TLC (*p* = 0.21) were able to significantly differentiate patients according to survival, with the strongest predictor being the TLCO (*p* = 0.0004). Of the PET-derived biomarkers, TBR was a significant predictor of patient survival (*p* = 0.019); curves are shown in Fig. 2. The synthetic score generated to express the total burden of parenchymal damage evaluated by qCT was significantly predictive of patient outcome (*p* = 0.0013); however, the strongest predictor of patient outcome from the qCT parameters was the vessel percentage (*p* = 0.0001; Fig. 3).

The KM analyses were then repeated using the optimal cutoff as threshold for the groups’ separation. Optimal cutoff and corresponding median survival are shown in Table 4. SUV_max_ and SUV_min_ were confirmed to be non-predictive of patient outcome (*p* = 0.081 and *p* = 0.12 respectively). Patients with TBR < 4.8 had a median survival of 70.6 months as compared to 26.5 months in patients with TBR > 4.8 at the baseline PET (*p* = 0.018). The synthetic score of parenchymal damage demonstrated a slightly better performance than TBR; patients with values <18.5 had a median survival of 70.6 months as compared to 22.2 months in patients that presented a higher burden of parenchymal damage (*p* = 0.0002).

**Table 4:** Median survival obtained from Kaplan–Meier analysis conducted using the optimal cutoff to discriminate between the two groups (presence or lack of event during follow-up)

Parameter	Cutoff	Median survival		*p* value
		< Cutoff	> Cutoff	
Pulmonary function tests				
FVC	72	26.37	86.47	0.0044
FEV1	96.3	35.37	70.6	0.02
TLC	65.6	28.57	86.73	0.027
KCO	50	19.9	46.8	0.00097
TLCO	44.8	23.53	70.60	0.00027
GAP index	4	70.6	19.9	0.00046
PET-derived parameters				
SUV_max_	2.4	86.73	34.43	0.081
SUV_min_	0.6	26.53	67.50	0.12
TBR	4.8	70.60	26.53	0.018
qCT				
Total volume (cm^3^)	4352.77	28.57	80.93	0.011
Vessel percentage (%)	3.66	86.47	19.90	<0.0001
Normal parenchyma (%)	80.20	26.37	86.37	0.00029
Hyperlucent (%)	11.12	46.80	22.23	0.0038
Ground-glass (%)	6.79	67.5	16.3	<0.0001
Reticular (%)	10.96	70.60	26.53	0.004
Honeycomb (%)	4.23	40.80	16.63	0.0022
Parenchymal damage (%)	18.48	70.60	22.23	0.0002
Synthetic scores				
GAP + PET	6	43.90	11.56	<0.0001
GAP + PET + qCT	6	86.47	17.23	<0.0001

FVC = forced vital capacity, FEV1 = forced expiratory volume in 1 s, TLC = total lung capacity, KCO = carbon monoxide transfer coefficient, TLCO = transfer factor of the lung for carbon monoxide

The parameters that significantly predicted outcome with KM analysis were used as the input for the Cox multivariate analysis and the Cox forward stepwise regression analysis; results are shown in Table 5. The stepwise regression confirmed that TBR is an independent prognostic predictor (*p* = 0.01). Moreover, in a model constructed including the qCT parameters, the forward stepwise Cox regression analysis confirmed that the volume of normal parenchyma, the vessels, and the hyperlucent pattern are independent prognostic indicators (*p* = 0.03, *p* = 0.01 and *p* = 0.01, respectively).

**Table 5:** Cox modelling of survivals according to pulmonary function tests, TBR, and qCT parameters

Parameter	Hazard ratio (95% CI)	*p* value
Pulmonary function tests		
FVC	0.99 (0.90–1.11)	0.997
FEV1	0.99 (0.91–1.07)	0.775
TLC	0.94 (0.86–1.02)	0.155
KCO	0.96 (0.90–1.02)	0.146
TLCO	0.99 (0.87–1.12)	0.849
GAP index	3.77 (1.12–12.69)	0.032
PET-derived parameters		
TBR	1.09 (1.00–1.19)	0.043
qCT		
Total volume (cm^3^)		
Vessel percentage (%)	2.57 (1.04–6.38)	0.0414
Normal parenchyma (%)	5.22 (4.75–5.73)	0.9202
Hyperlucent (%)	5.12 (4.63–5.67)	0.9211
Ground-glass (%)	4.76 (4.32–5.25)	0.9246
Reticular (%)	4.74 (4.32–5.20)	0.9249
Honeycomb (%)	5.00 (4.57–5.46)	0.9223
Parenchymal damage (%)	1.09 (0.99–1.19)	0.008

FVC = forced vital capacity, FEV1 = forced expiratory volume in 1 s TLC = total lung capacity, KCO = carbon monoxide transfer coefficient, TLCO = transfer factor of the lung for carbon monoxide

### Modified GAP score

**Fig. 4:**
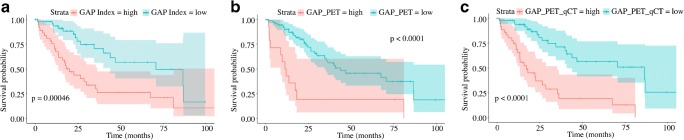
Kaplan–Meier plots of survival analysis. Patients were classified according to the optimal cutoff values as described in the methods section; *a* represents the GAP index, *b* is GAP_PET (obtained from the addition of GAP and TBR), and *c* is GAP_PET_qCT (obtained adding the qCT to the GAP_PET); additional details on the synthetic score can be found in the methods section. The log-rank test demonstrated that all the scores were statistically significant predictors of survival (*p* values in corresponding panel); however, adding information from PET and qCT improved the ability to differentiate between a better and worse prognosis

The results of the KM analysis conducted on the incremental scores (GAP_PET and GAP_PET_qCT) obtained by modifying the GAP score are presented in Fig. 4. The two incremental scores were all significantly able to predict patient survival (*p* < 0.0001); however, their ability to predict median survival differed (Table 6). Using an optimal cutoff of 6, the GAP_PET showed a median survival of 43.9 months in patients with a score lower than the cutoff, and median survival of 11.6 months in patients above the cutoff. The GAP_PET_qCT score showed an improvement in outcome prediction, particularly in the worst outcome group, with a median survival of 86.5 months in patients with a score lower than 6, and median survival of 17.2 months in patients with a score above the cutoff.

**Table 6:** Distribution of patients (expressed as n) according to the risk category as defined by GAP index, GAP PET values, and quantitative CT parameters and their corresponding average survival (expressed in months)

Scoring system	Low risk		Intermediate risk		High risk	
	Patients (*n*)	Survival (months)	Patients (*n*)	Survival (months)	Patients (*n*)	Survival (months)
GAP index	31	33.3	38	29.3	21	24.4
GAP index + PET	41	35.0	36	27.6	14	19.5
GAP index + PET + qCT	54	36.3	25	21.8	12	16.3

## Discussion

Our study has shown that in IPF patients, the baseline measures of FDG PET and several qCT parameters in HRCT are potential independent biomarkers related to patient survival. Their combined use can have a synergistic effect in the assessment of disease prognosis. These data demonstrate that in IPF patients, both pulmonary glucose uptake and qCT are independent prognostic factors. Moreover, these factors are synergistic and may offer better outcome modeling than current GAP analysis alone. This is potentially important in IPF patients, where there is a lack of validated biomarkers for risk stratification and therapeutic intervention.

Among the PET parameters, patients with a high TBR had a worse prognosis, while for the qCT, the percentage of normal parenchyma and the percentage of vessels (higher % corresponding to worse prognosis) were the strongest independent imaging biomarkers of survival (Table 4). These findings confirm data previously reported elsewhere [13].

The unexpected signal provided by pulmonary vessel volume (PVV) in this study has been described but is not fully comprehensible. Jacob et al. provided three plausible explanations: 1) blood-flow diversion from advanced fibrotic areas to relatively spared lung regions, with aberrant dilatation of capacitance vessels resulting in increased PVV; 2) dilatation effect on blood vessels of increased negative pressure during inspiration due to increased lung stiffness in IPF patients; and 3) the effect of pleuroparenchymal and bronchopulmonary arterial anastomosis [14].

Further explanations have been also proposed, including the presence of vascular abnormalities in fibrotic lungs, demonstrated histologically and reminiscent of pulmonary venous occlusive disease with aberrant capillary duplication. The reported increased vascular volume was found in less affected areas of the lung and correlated with pulmonary arterial pressure as estimated by transthoracic echocardiography [15].

It is interesting that PET is able to detect subtle metabolic changes in visually normal or minimally involved lung in patients with IPF [16]. In this regard, the combined application of metabolic, morphological, and quantitative information may enable more accurate assessment of early disease, for example, to determine whether the subtle changes seen on FDG PET are in fact due to regional increases in blood flow in areas of limited interstitial lung pathology.

When we combined the pulmonary PET uptake, qCT, and PFT parameters, we found that the combination of these three independent parameters had the strongest association with survival. In fact, even in IPF patients with good PFTs, the pulmonary ^18^F-FDG uptake and the extent of morphological abnormalities on HRCT might help identify subpopulations of patients that had a poorer outcome [8]. Using qCT and PET, we created two modified versions of the GAP score that improved the capacity to classify patients according to their outcome at the follow-up using baseline tests. This may have relevance in the clinical setting in determining treatment recommendations based on a combination of the three independent variables (GAP + qCT + PET).

The synergies between GAP, PET, and qCT are encouraging. In GAP stage I, we identified IPF patients with a worse outcome than the other GAP I patients, and they may have benefited from treatment. Likewise, there were patients in GAP stage II and particularly III that showed lower ^18^F-FDG uptake and qCT parameters, whose outcome was more favorable. This may have important clinical implications, as with our data we were able to re-classify many patients from GAP III into the new modified GAP I score despite impaired lung function (FVC < 80% predicted), compared to a group of patients with mildly impaired lung function (FVC > 80% predicted) that progressed rapidly. Thus, the synergistic use of ^18^F-FDG PET and qCT in this context raises the possibility for more accurate selection of patients that may benefit most from pirfenidone or nintedanib treatment from a wider patient population.

HRCT is the main diagnostic imaging tool in IPF, but until recently there have been only limited data on the prognostic use of this imaging technique. This may be because in many cases, the morphological appearance does not allow for accurate and reproducible assessment [17]. However, a number of studies investigating the association between mortality and different variables, including normal lung, centrilobular emphysema, and number of vessels, have shown qCT to be superior to visual HRCT scoring [18]. qCT-derived features have also outperformed visual CT patterns in predicting outcome across several fibrosing lung diseases other than IPF.

One of the best known software algorithms, CALIPER (Computer-Aided Lung Informatics for Pathology Evaluation and Rating), based on lung texture analysis, was developed at the Biomedical Imaging Resource, Mayo Clinic, Rochester, MN, USA. This software has been shown to be reproducible and robust across a wide variety of acquisition and reconstruction techniques, including low- and ultralow-dose (0.1–0.3 mSv) CT techniques with both filtered back-projection and iterative reconstruction. The model proposed in our study is based on CALIPER and provides a detailed map of lung textures that are key to identifying ILDs and other fibrotic conditions [18]. Jacob et al. recently reported their application of CALIPER quantitative HRCT in IPF patients, showing that stratification using CALIPER variables and PFTs provided a stronger mortality signal than stratification using the GAP index alone [13].

The technical protocol we have developed in this study is novel and uses a low-dose HRCT acquired in breath-hold at the end of half-dose FDG PET. This combined quantitative approach may pave the way for more detailed longitudinal studies. However, we recognize that there are limitations: several technical factors related to qCT and PET need to be improved. Regarding qCT, several analytical methods have been described (e.g. segmentation and feature extraction based on lung density [measured in Hounsfield units]), all heavily influenced by CT dose, slice thickness, and reconstruction kernels. In our study, slightly different HRCT acquisition protocols adopted at the start of our recruitment since 2006 led to the exclusion of some patients due to different reconstruction methods or scanning protocols.

Limitations of PET imaging also need to be recognized, such as the importance of air and tissue fraction and motion correction. For example, in the normal lung, previous studies showed that the uptake distribution without air fraction correction (AFC) appeared uniform throughout the lung, but on correcting for the air component, the results for the regional uptake changed [19, 20]. Also, future studies need to explore the use of texture heterogeneity analysis as part of the overall/comprehensive qCT analysis techniques, which could provide additional insight into morphological feature extraction [21].

We also acknowledge that the imaging was not always performed at the time of diagnosis, which is a common problem for imaging studies, as patients often present at later stages of the disease.

We recognize that our combined approach is not feasible for all patients, as it is time-consuming and is not always financially justifiable. On the other hand, current therapies for IPF are expensive and often limited by side effects, and not all patients may benefit from them. There are no validated disease response biomarkers available, and our combined approach may refine the stratification of this heterogenous group of patients, as well as speed the assessment of novel therapies, and enable a personalized approach in selected cases.

Finally, although the PET and HRCT acquisition scans can be done on different scanners and the results of the two techniques evaluated independently, some newer PET/CT scanners allow simultaneous HRCT/PET acquisition in breath-hold, reducing the time for a double scan and limiting ionizing radiation.

## Conclusion

We have shown that high pulmonary uptake of ^18^F-FDG and several qCT parameters are associated with mortality in patients with IPF. These PET and qCT findings can be used synergistically with PFTs and could help offer a personalized approach to treatment for individual patients.
